# miR-1915-3p regulates megakaryocytic and erythroid differentiation by targeting SOCS4

**DOI:** 10.1186/s12959-024-00615-6

**Published:** 2024-08-09

**Authors:** Xin Yuan, Pengcong Liu, Lei Xu, Liqing Liang, Qian Dong, Tao Fan, Wen Yue, Mingyi Qu, Xuetao Pei, Xiaoyan Xie

**Affiliations:** Stem Cell and Regenerative Medicine Lab, Beijing Institute of Radiation Medicine, No. 27 Taiping Road, Haidian District, Beijing, 100850 China

**Keywords:** Megakaryocytic and erythroid differentiation, miR-1915-3p, SOCS4

## Abstract

**Background:**

Proper control of the lineage bias of megakaryocytic and erythroid progenitor cells (MEPs) is of significant importance, the disorder of which will lead to abnormalities in the number and function of platelets and erythrocytes. Unfortunately, the signaling pathways regulating MEP differentiation largely remain to be elucidated. This study aimed to analyze the role and the underlying molecular mechanism of miR-1915-3p in megakaryocytic and erythroid differentiation.

**Methods:**

We utilized miRNA mimics and miRNA sponge to alter the expression of miR-1915-3p in megakaryocytic and/or erythroid potential cells; siRNA and overexpression plasmid to change the expression of SOCS4, a potential target of miR-1915-3p. The expression of relevant surface markers was detected by flow cytometry. We scanned for miR-1915-3p target genes by mRNA expression profiling and bioinformatic analysis, and confirmed the targeting by dual-luciferase reporter assay, western blot and gain- and lost-of-function studies. One-way ANOVA and t-test were used to analyze the statistical significance.

**Results:**

In this study, overexpression or knockdown of miR-1915-3p inhibited or promoted erythroid differentiation, respectively. Accordingly, we scanned for miR-1915-3p target genes and confirmed that SOCS4 is one of the direct targets of miR-1915-3p. An attentive examination of the endogenous expression of SOCS4 during megakaryocytic and erythroid differentiation suggested the involvement of SOCS4 in erythroid/megakaryocytic lineage determination. SOCS4 knockdown lessened erythroid surface markers expression, as well as improved megakaryocytic differentiation, similar to the effects of miR-1915-3p overexpression. While SOCS4 overexpression resulted in reversed effects. SOCS4 overexpression in miR-1915-3p upregulated cells rescued the effect of miR-1915-3p.

**Conclusions:**

miR-1915-3p acts as a negative regulator of erythropoiesis, and positively in thrombopoiesis. SOCS4 is one of the key mediators of miR-1915-3p during the differentiation of MEPs.

**Supplementary Information:**

The online version contains supplementary material available at 10.1186/s12959-024-00615-6.

## Background

Megakaryocytic and erythroid progenitor cells (MEPs) are bipotent hematopoietic progenitors, capable of differentiating into the megakaryocytic lineage to give rise to megakaryocytes and platelets, and into the erythroid lineage to generate erythrocytes. The regulation of the differentiation tendency of MEPs is of significance since disorders in MEP development will lead to abnormalities in the number and function of platelets and erythrocytes. For example, in patients with advanced tumors, MEPs tend to differentiate towards the megakaryocytic lineage, leading to anemia and thrombocytosis, which will result in thrombosis and tumor metastasis, subsequently a poor prognosis [1]. It has been reported that the differentiation of MEPs is regulated by transcription factors such as GATA-1, GATA-2, RUNX-1, and KLF1 [2–6].

MicroRNAs (miRNAs) are small non-coding single-stranded RNA molecules, that target complementary sequences in mRNAs, leading to posttranscriptional degradation or inhibition of gene expression. It has been proven miRNAs play an important role in diverse cell biological and physiologic processes [7, 8]. Previous reports [9–12] and our studies [13–15] have demonstrated that there are many miRNAs regulate hematopoietic lineage commitment, including erythropoiesis and thrombopoiesis, some of them, such as miR-144, miR-27a, miR-9, are even involved in both parts of megakaryocytic and erythroid differentiation. For MEP regulation, miR-150 promotes the differentiation of MEPs to the megakaryocytic lineage and inhibits their differentiation to the erythroid progenitors [16] and miR-125b increased proliferation and self-renewal of MEPs and megakaryocyte progenitors [17]. However, the functions and mechanisms of miRNAs in erythroid and megakaryocytic differentiation are still not well elucidated. The further elaboration of new miRNAs and their targets in the manipulation of cell fate will be helpful to understand pathogenesis and potential drug targets of human diseases in which the number and function of platelets or erythrocytes are abnormal, such as hypothrombocytopenia, anemia, and polycythemia vera.

It was reported previously that microparticles (MPs) derived from megakaryocytes and platelets constitute ∼ 70–90% of circulating MPs, and MPs are major transport vehicles for various miRNAs. A few reports and our study demonstrated that activated platelets, through deriving MPs, enrich and transfer genetic information to target cells to modulate cellular gene expression and activity [18–20]. One of the miRNAs identified in platelet-derived microparticles (PMPs), the miR-126, is known to modify the transcriptome of macrophages and reprogram their function towards a phagocytic phenotype [21]. Another miRNA, miR-223, being abundant in platelets and PMPs, attenuates TNF-α induced monocyte adhesion to arterial endothelium by targeting ICAM-1, it is also reported by our previous study that miR-223 modulates megakaryocyte polyploidization via targeting MYH10 [15, 22, 23].

We have discovered that miR-1915-3p was highly enriched in human PMPs and able to be transported to hematopoietic stem/progenitor cells (HSPCs) to drive the cells toward a megakaryocytic fate and produce platelets. Overexpression of miR-1915-3p in HSPCs increased the count of MEPs and megakaryocytic cells, instead of erythroid cells. Accordingly, we hypothesized that miR-1915-3p might play a role in the differentiation of MEPs [20]. In this study, we systematically identified that miR-1915-3p promoted the hematopoietic differentiation to the megakaryocytic lineage and inhibited the differentiation to erythroid progenitors, one of the targets that mediate its function was SOCS4.

## Materials and methods

### Purification of human cord blood mononuclear cells (CB-MNCs) and CD34^+^ cells

Human cord blood samples were obtained from the umbilical cord after the delivery of normal pregnancies with the patient’s informed consent. The studies involving human subjects were approved by the Ethical Committee of the Beijing Institute of Radiation Medicine and the permit number is AF/SC-08/028. Mononuclear cells were isolated by Ficoll-Hypaque density gradient centrifugation (1.077 g/L; TBDscience, Tianjin, China). Subsequently, MNCs were magnetically labeled with human CD34 MicroBead Kit (Miltenyi Biotec) and CD34^+^ cells were isolated from MNCs by magnetic force following the manufacturer’s instructions (Miltenyi Biotec).

### Cell culture and differentiation

Human erythroleukemia cell line K562 cells and human megakaryoblastic leukemia cell line Meg-01 cells were cultured in RPMI-1640 medium (Gibco) supplemented with 10% fetal bovine serum (FBS, AusGenex). Human megakaryoblastic leukemia cell line UT-7 cells were maintained in RPMI-1640 medium supplemented with 10% FBS and 5 U/mL EPO (PeproTech). Human erythroleukemia cell line TF-1 cells were maintained in RPMI-1640 medium supplemented with 10% FBS and 5 ng/mL GM-CSF (PeproTech). For megakaryocytic differentiation, K562, Meg-01 and UT-7 cells were treated with 2 nM PMA (Sigma-Aldrich) for 3 days. For erythroid differentiation, K562 cells were treated with 40 µM Hemin (Selleck) and 0.1 µg/mL Ara-C (Sigma-Aldrich) for 4 days and then continued to be treated with 40 µM Hemin and 210 mM DMSO (Sigma-Aldrich) for 4 days to induce enucleation. TF-1 cells were cultured in RPMI-1640 medium supplemented with 10% FBS and 5 U/mL EPO instead of GM-CSF to induce erythroid differentiation. Human CB-MNCs and CD34^+^ cells were grown in erythroid differentiation medium for 5 ∼ 14 days, which is based on StemSpan SFEM II Medium (STEMCELL Technologies) supplemented with a cytokine cocktail containing 5 U/mL EPO, 100 ng/mL SCF, 40 ng/mL IGF-1 (PeproTech), 100 µg/mL holo-Transferrin human (Sigma-Aldrich), 2mM GlutaMAX™ Supplement (Gibco) and 40 µg/mL CD Lipid Concentrate (Gibco). All these cells were cultured at 37 ^o^C in a humidified atmosphere with 5% CO_2_.

### Cell transfection

Lipofectamine 2000 (Invitrogen, CA, USA) was utilized for cell transfection. Briefly, 2 × 10^5^ leukemia cells or 1 × 10^6^ primary cells were seeded in 6-well plates and transfected with 1.6 μg per well miRNA mimics or siRNA (Genepharma, Shanghai, China), or 4 µg per well plasmid DNA, according to the manufacturer’s instructions. Considering the low efficiency and long time-consuming for stable plasmid transfection in primary hematopoietic stem progenitor cells, for gene expression modification, MNCs were transfected with miRNA mimics or siRNA, and human leukemia cell lines, K562, Meg-01, TF-1 and UT-7 cells, with high stability during in vitro culture, were usually transfected with plasmid. Especially, siRNAs mimics against SOCS4, were transfected to knockdown human SOCS4 mRNAs in all cells. Cells were incubated with transfection mixtures for 6 h and then transferred to a fresh medium for the subsequent studies. Fluorescently labeled (FAM) negative control siRNA was used to analyze the stability and transfection efficiency of the siRNA.

To manipulate miRNA expression in MNC cells, miR-1915-3p mimics (miR-1915) and negative control (NC) miRNAs were transfected into MNC cells, The miR-1915 was a double-stranded RNA with the sequence 5′- CCCCAGGGCGACGCGGCGGG-3′ for the sense strand and 5′- CGCCGCGUCGCCCUGGGGUU-3′ for the antisense strand. To knockdown human SOCS4 mRNAs in cell lines, specific siRNAs targeting the coding region were designed. The SOCS4 siRNA was a double-stranded RNA with the sequence 5′- CAGACUUGUCUC AGACUGATT-3′ for the sense strand and 5′- UCAGUCUGAGACAAGUCUGTT- 3′ for the antisense strand. The negative control was a double-stranded RNA with the sequence 5′- UUCUCCGAACGUGUCACGUTT-3′ for the sense strand and 5′- ACGUGACACGUUCGGAGAATT-3′ for the antisense strand.

Stable miR-1915-3p overexpressing cell lines were developed using the recombinant plasmid (pcDNA3.0-pri-miR-1915-3p-neomycin), and miR-1915-3p downregulated cell lines were built with pcDNA3.0-miR-1915-3p-sponge-neomycin plasmid [20]. Stable SOCS4 overexpressing cell lines were developed using the recombinant plasmid (pcDNA3.0-SOCS4-neomycin). The empty vector (pcDNA3.0-neomycin) was used as a transfection control which carries the neomycin resistance gene as a selection marker. After cell transfection for 48 h, the cells were transferred to a fresh medium in the presence of 500 µg/mL G418 for screening stable-transfected cell lines.

### Antibodies and staining reagents

The following antibodies were used for either cell sorting or flow cytometry analysis: directly conjugated phycoerythrin (PE) anti-human CD235a (BD biosciences); allophycocyanin (APC) anti-human CD71 (BD biosciences); BV605 anti- human CD71 (BD biosciences); fluorescein isothiocyanate (FITC) anti-human CD41 (eBioscience, San Diego, CA, USA); PE anti- human CD41 (eBioscience); APC anti- human CD61 (eBioscience); APC anti- human CD62P (eBioscience); PE-, APC- and FITC-conjugated immunoglobulin G1 mAb (obtained from eBioscience) were used as isotype controls. The Fixable Viability Stain 510 was purchased from BD Biosciences. The nucleic acid stain SYTO 16 was purchased from Thermo Fisher Scientific, and DRAQ5 was purchased from Cell Signaling Technology.

Antibodies against SOCS4 used for Western blot were purchased from R&D Systems (MAB5628, 1:2000). The HRP-conjugated GAPDH antibodies (AC035, 1:5000) and goat anti-mouse HRP-conjugated secondary antibody (AS003, 1:5000) was purchased from ABclonal. GAPDH was used as a loading control.

### Flow cytometric analysis and fluorescence-activated cell sorting (FACS)

Cells were collected at indicated time points (induced to megakaryocytic or erythroid cells) and washed with PBS, subsequently labeled with monoclonal antibodies in PBS for 30 min at 4 °C and washed twice before analysis by BD FACSAria II flow cytometer (BD Biosciences). FlowJo software (Version 10, TreeStar) was utilized to analyze the expression of erythroid cell surface markers CD71 and CD235a, and megakaryocyte surface markers CD41 and CD61. For enucleation analysis, cells were stained with SYTO 16 or DRAQ5 after staining of cell surface markers.

On day 14 of erythroid induction, cells were harvested, stained and sorted by using anti-human CD71-APC and CD235a-PE antibodies [13]. On day 6 of megakaryocytic differentiation, cells were sorted by using anti-human CD61-APC and CD41-PE antibodies [24]. The sorting was performed by using BD FACSAria II flow cytometer (BD Biosciences) and FACS Diva Software (Version 7, BD Biosciences).

### RNA isolation and reverse transcription‑quantitative PCR (RT-qPCR)

As described in the manufacturer’s instructions, total RNA was extracted from the transfected cells or induced cells using TRIzol reagent (Invitrogen, Thermo Fisher Scientific) and quantified using an ultra‑microspectrophotometer Nanodrop 2000c (Thermo Fisher Scientific) at 260 nm. For RNA processing, ReverTra Ace® qPCR RT Master Mix (TOYOBO) was used for reverse transcription of 1 µg total RNA into cDNA. For miRNA processing, miScript II RT Kit (QIAGEN) was used for reverse transcription into cDNA. THUNDERBIRD^®^ Next SYBR™ qPCR Mix (TOYOBO) and QuantiNova SYBR Green PCR Kit (QIAGEN) for miRNAs quantitation were used in RT-qPCR analysis. RT-qPCR was performed by CFX Connect™ Real-Time PCR Detection System (Bio-Rad) and data was analyzed by Bio-Rad CFX Manager software (Bio-Rad) with three replicates set for each reaction. Since RNU6B (U6) is a frequent internal control for miRNA assay and used for data normalization of miRNAs in previous reports [14, 15, 20, 25, 26], miR-1915-3p expression was normalized to U6, while other human genes were normalized to GAPDH. The 2^-ΔΔCT method was used to analyze the relative expression level. The sequences of the RT-qPCR primers used for the experiments are shown in Supplementary Table 1.

### mRNA expression profiling and miRNA target prediction

mRNA expression profiling and miRNA target prediction have been reported previously [20]. And novel microarray data were deposited to the Gene Expression Omnibus (https://www.ncbi.nlm.nih.gov/geo/) under accession number GSE152078 and GSE152079. The expression of mRNA was log-2 transformed after quantile normalization. Differentially expressed mRNAs (> 1.2-fold change or < 0.833-fold change) in cells treated with PMPs were selected for further bioinformatics analysis.

### Dual-luciferase reporter assay

The cloned sequences and putative binding sites of miR-1915-3p on SOCS4 3′-UTR are demonstrated in Fig. 2. The sequences were cloned into the pGL3 control vector (Promega, Madison, WI, USA). Stable miR-1915-3p overexpressing K562 cells (2 × 10^5^) were co-transfected with 800 ng pGL3-wildtype-SOCS4 vector (or pGL3-mutation-SOCS4 vector or pGL3 control vector) and 2 ng pRL Renilla luciferase vector (Promega) using Lipofectamine 2000 (Invitrogen). After 48 h of transfection, firefly luciferase activity was measured by using the Dual-Luciferase reporter assay system (Promega) according to the manufacturer’s instructions and normalized to Renilla luciferase activity. The experiments were independently repeated three times.

### Western blot analysis

Total proteins were extracted with RIPA lysis buffer containing proteinase inhibitors from transfected cells and quantified using the Pierce™ BCA Protein Assay Kits (Thermo Fisher Scientific). The same amount of protein was separated by SDS-PAGE and transferred to a polyvinylidene difluoride (PVDF) membrane. The membrane was blocked with 5% non‑fat milk in TBS/Tween‑20 (0.1%) for 1 h at room temperature, and incubated overnight at 4 ^o^C with the primary antibody against SOCS4 (MAB5628, 1:2000, R&D Systems). After washing, the membrane was incubated with goat anti-mouse HRP-conjugated secondary antibody (AS003, 1:5000, ABclonal) for 1 h at room temperature and developed using an Immobilon Western Chemiluminescent HRP Substrate reagent (EMD Millipore, Sigma-Aldrich). Anti-GAPDH antibody (AC035, 1:5000, ABclonal) was used as an internal reference. Blots were imaged with the Amersham Image 680 and quantitatively analyzed by ImageJ.

### Immunohistochemical (IHC) staining

A mouse carbon tetrachloride (CCl_4_) mediated acute liver injury (ALI) model was established previously [20]. Bone marrow samples were harvested on day 8 post injury and embedded in paraffin after fixation in 10% formalin for hematoxylin-eosin staining and DAB staining. SOCS4 protein expression in the bone marrow was assessed by the proportion of SOCS4-DAB positive cells/hematoxylin-eosin staining cells, using TissueFAXS and TissueQuest 6.1 software (TissueGnostics).

### Benzidine staining

Benzidine (Sigma) staining was used to identify hemoglobin-containing cells. 10 µL of 30% hydrogen peroxide was added to 1 mL of benzidine immediately before use. A total of 5 × 10^4^ cells were centrifuged onto a slide and fixed with methanol (-20 ^o^C) for 2 min, following which benzidine-hydrogen peroxide mixture (50 µL) was added and incubated for 1 h at room temperature. Subsequently, cells were stained with Wright’s‑Giemsa and observed by using an upright microscope (ECLIPSE Ni series, Nikon). Benzidine-positive cells were stained dark blue, and the proportion were analyzed by using TissueFAXS and TissueQuest 6.1 software (TissueGnostics).

### Wright’s‑Giemsa staining

A total of 5 × 10^4^ cells were centrifuged onto a slide. After drying at room temperature, the staining solution A (Wright’s‑Giemsa Staining Solution) was added for 1 min and the staining solution B (PBS) was added and mixed for 10 min at room temperature. After rinsing the excess dye with running water, the cells were observed under an upright microscope (ECLIPSE Ni series, Nikon).

### Statistical analysis

Statistical analysis was conducted on data from three or more biologically independent experimental replicates using GraphPad Prism 8 (GraphPad Software, Inc.). All data are shown as the mean ± standard error of means (SEM) unless otherwise indicated. Data comparisons between two groups were conducted by Student’s t-test and those among multiple groups by one-way ANOVA. A p-value less than 0.05 is considered significant. *, *p* < 0.05; **, *p* < 0.01; ***, *p* < 0.001.

## Results

### miR-1915-3p is involved in the regulation of erythroid differentiation

**Fig. 1:**
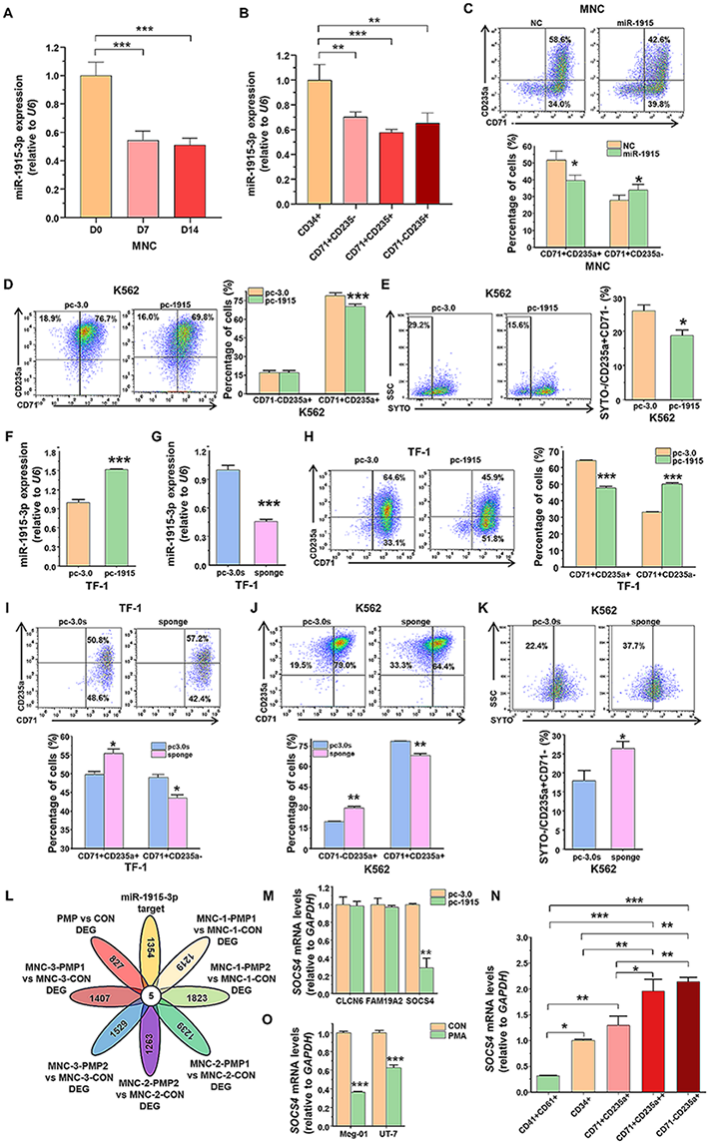
Role of miR-1915-3p in erythroid differentiation and target genes identification. (**A**) Mononuclear cells (MNCs) were induced toward erythrocytes. The endogenous expression of miR-1915-3p during erythropoiesis on Days 0, 7 and 14 was determined by RT-qPCR. (**B**) In CD34^+^ HSPCs at erythroid induction day 14, erythroblasts were stained and sorted by CD71/CD235a antibodies. miR-1915-3p expression was detected by RT-qPCR in different erythroid developmental stage cells. (**C**) Flow cytometry analysis of MNC erythroid differentiation with miR-1915-3p overexpression. MNCs were transiently transfected with miR-1915-3p mimics or negative control (NC) and cultured in an erythroid differentiation medium, flow cytometry was used to examine the expression of surface markers CD71 and CD235a on induction day 5. (**D**, **E**) Flow cytometry analysis of K562 cell erythroid differentiation with miR-1915-3p overexpression. Stable miR-1915-3p overexpressed K562 cells were induced to erythroid differentiation and enucleation, flow cytometry was used to examine the expression of CD71 and CD235a (**D**) and the proportion of enucleated cells (SYTO 16 negative cells, **E**). (**F**, **G**) RT-qPCR analyzed miR-1915-3p expression in TF-1 cells. TF-1 cells were stably transfected with a miR-1915-3p (**F**) or miR-1915-3p sponge (**G**) recombinant plasmid. (**H**, **I**) Flow cytometry analysis of TF-1 cell erythroid differentiation with miR-1915-3p overexpression or downregulation. The stably transfected miR-1915-3p (**H**) or miR-1915-3p sponge (**I**) recombinant plasmid TF-1 cells were induced to erythroid differentiation and the expression of CD71 and CD235a were examined by flow cytometry. (**J**, **K**) Flow cytometry analysis of K562 cell erythroid differentiation with miR-1915-3p downregulation. Stable miR-1915-3p-sponge-transfected K562 cells were induced to erythroid differentiation and enucleation, flow cytometry was used to examine CD71 and CD235a (**J**) and the proportion of enucleated cells (SYTO 16 negative cells, **K**). (**L**) Venn diagram demonstrates the overlap between the differentially expressed genes (DEGs) of PMP treatment in mRNA expression profiling analysis and the potential target genes of miR-1915-3p. (**M**) RT-qPCR was used to analyze the potential common target gene expression in K562 cells. (**N**) Endogenous expression of SOCS4 was detected by RT-qPCR during megakaryocytic and erythroid differentiation. (**O**) SOCS4 expression was detected by RT-qPCR in Meg-01 and UT-7 cells under megakaryocytic induction. Data are presented as mean ± SEM

Previous studies have found that endogenous expression of miR-1915-3p increases during megakaryocytic differentiation and platelet production and miR-1915-3p promotes megakaryocytic differentiation and platelet production [20]. However, the expression during erythropoiesis and the effect of miR-1915-3p on erythroid differentiation is unknown. We have previously sorted erythrocytes at each differentiation and maturation phases by the expression levels of CD71 and CD235a and confirmed by the morphology of the cells [13]. To verify the dynamics of miR-1915-3p during erythroid differentiation, the expression of miR-1915-3p in phases of maturation was detected by RT-qPCR. miR-1915-3p was gradually down-regulated during primary cell erythropoiesis, suggesting that miR-1915-3p might be involved in erythroid differentiation (Fig. 1A, B). To determine whether miR-1915-3p regulates erythroid differentiation of primary human hematopoietic stem and progenitor cells, miR-1915-3p expression was modified in human CB-MNCs. The cells were transfected with miR-1915-3p mimics or negative control (NC) and cultured in an erythroid differentiation medium. We evaluated the effect of miR-1915-3p overexpression on erythroid surface markers. After 5 days of erythroid induction, miR-1915-3p mimics transfected MNCs showed a lower CD71^+^CD235a^+^ percentage than NC cells (Fig. 1C).

To further evaluate the effect of miR-1915-3p on erythroid differentiation, we overexpressed miR-1915-3p in K562 and TF-1 cells. K562 and TF-1 cells were stably transfected with pcDNA3.0-pri-miR-1915-3p-neomycin or pcDNA3.0-neomycin plasmid, then induced to erythroid differentiation and enucleation. Compared to the control group, the percentage of CD71^+^CD235a^+^ cells in K562 cells was significantly reduced (Fig. 1D). The enucleated cells (SYTO 16^−^/CD235a^+^ cells) in miR-1915-3p overexpression group were also decreased than the control group (Fig. 1E). The overexpression of miR-1915-3p in TF-1 cells was confirmed with RT-qPCR, and it reduced the CD71^+^CD235a^+^ percentage consistently with the effect on K562 cells (Fig. 1F, H).

Subsequently, K562 and TF-1 cells were stably transfected with pcDNA3.0-miR-1915-3p-sponge-neomycin plasmid or pcDNA3.0-sponge-neomycin control plasmid to determine whether the downregulation of miR-1915-3p contributes to erythroid differentiation. By using miR-1915-3p sponge, miR-1915-3p expression was successfully downregulated in TF-1 cells (Fig. 1G) and the percentage of CD71^+^CD235a^+^ was significantly increased (Fig. 1I). miR-1915-3p inhibition also elevated the percentage of CD71^−^CD235a^+^ cells and enucleation ratio (SYTO 16^−^/CD235a^+^ cells) in K562 cells (Fig. 1J, K). The above discoveries suggested that miR-1915-3p was involved in erythroid differentiation, besides functioning in megakaryocytic differentiation regulation.

### SOCS4 might be the miR-1915-3p target gene by bioinformatics analysis

To clarify how miR-1915-3p plays a regulatory role, the downstream target genes of miR-1915-3p were predicted by using three bioinformatics databases (PITA, microRNA.org and TargetScan). Totally 1354 potential target genes of miR-1915-3p were discovered through the prediction. On the other hand, it was known that miR-1915-3p was highly enriched in PMPs and could be transported to target cells by PMPs. We also performed a mRNA profiling microarray in CB-MNCs with or without PMP treatment. We reasoned that the target genes of miR-1915-3p in PMPs could be downregulated after PMP supplement. Subsequently, the intersection between bioinformatics-predicted miR-1915-3p targets and microarray-detected downregulated mRNAs identified 5 genes (CLCN6, FAM19A2, LSS, SCD and SOCS4) that might mediate the function of miR-1915-3p (Fig. 1L). SCD and LSS have been ruled out in our previous study with barely changes after miR-1915-3p upregulation from PMP addition [20]. To validate the finding, the mRNA levels of the other 3 genes were analyzed by RT-qPCR in the K562 cells stably transfected with pcDNA3.0-pri-miR-1915-3p-neomycin or pcDNA3.0-neomycin plasmid. As shown in Fig. 1M, in the 5 putative miR-1915-3p targets, only SOCS4 gene could be downregulation after miR-1915-3p overexpression. Thus, we ultimately narrowed our analysis of potential target genes of miR-1915-3p to SOCS4, which might be involved in the regulation of erythroid and megakaryocytic differentiation.

Then, we analyzed the endogenous expression of SOCS4 in erythroid and megakaryocytic differentiation. As we known, the expression of CD41 and CD61 increased during megakaryopoiesis, and the expression of CD71 and CD235a increased during erythrogenesis, while surface expression of CD34 in both were decreased. Using these well-established surface markers, we separated five populations of cells from human CB-MNCs derivatives by fluorescence activated cell sorting (FACS) technology. We refer to them as: CD34^+^ cells that representing HSPCs, CD41^+^CD61^+^ cells that representing megakaryocytes, and CD71^+^CD235a^+^, CD71^+^CD235a^++^ and CD71^−^CD235a^+^ cells that representing sequentially mature erythrocytes. Subsequently, the mRNA expression levels of SOCS4 were analyzed in these cells. The data showed that SOCS4 gene was relatively high expressed in erythrocytes and low expressed in megakaryocytes (Fig. 1N). Similarly, following PMA treatment, the expression of SOCS4 coincidently decreased during Meg-01 and UT-7 megakaryocytic induction (Fig. 1O). These results suggested that SOCS4 might be the miR-1915-3p target gene that mediates the function of miR-1915-3p.

### SOCS4 expression is directly regulated by miR-1915-3p

**Fig. 2:**
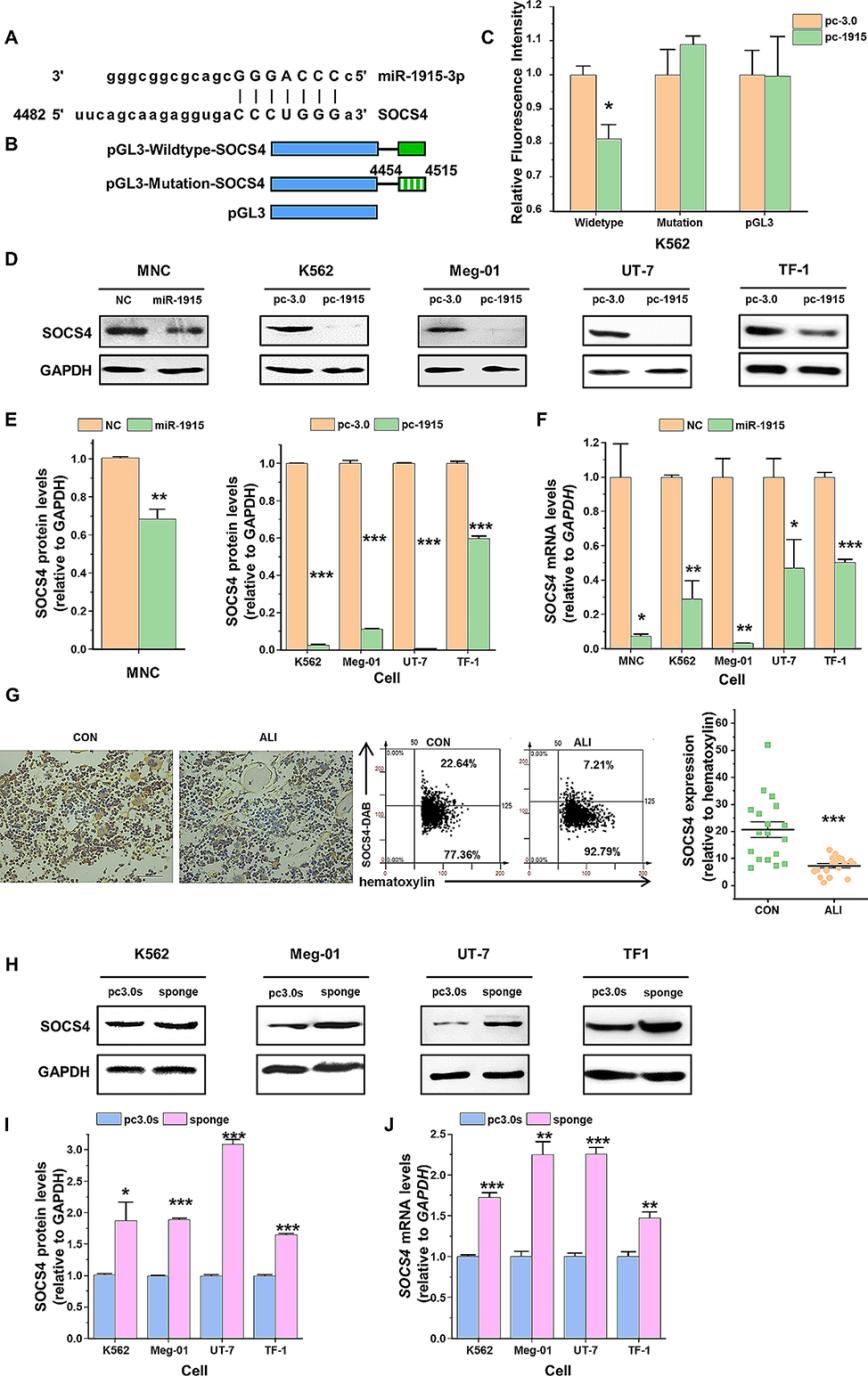
SOCS4 is one of the direct targets of miR-1915-3p. (**A**) Putative miR-1915-3p binding sequence in the SOCS4 3′-UTR. (**B**) pGL3-wildtype-SOCS4 vector (or pGL3-mutation-SOCS4 vector or pGL3 control vector) were transfected into stably established K562 cells with miR-1915-3p or pcDNA3.0 control vector overexpression. (**C**) Luciferase activity was measured and normalized by Renilla luciferase activity. (**D**-**F**) Detection of SOCS4 expression under miR-1915-3p upregulation. miR-1915-3p mimics or overexpression plasmid were transfected into the MNCs, K562, Meg-01, UT-7 and TF-1 cells. The protein levels of SOCS4 were analyzed by Western blot (**D**), and normalized by reference genes GAPDH (**E**). The mRNA levels of SOCS4 were examined by RT-qPCR (**F**). (**G**) IHC analysis of bone marrow in mice 8 days after CCl_4_ treatment, left: representative sections of treated tissue stained with mAb to SOCS4 (brown) and hematoxylin to nucleus (blue); right: the number of SOCS4 positive cells/the number of nucleated cells per high power field. The scale bar is 50 μm. The results are the average values of nine random views of two independent experiments. (**H**-**J**) Detection of SOCS4 expression under miR-1915-3p downregulation. miR-1915-3p sponge plasmid or pcDNA3.0-sponge control plasmid were transfected into the K562, Meg-01, UT-7 and TF-1 cells. The protein levels of SOCS4 were analyzed by Western blot (**H**), and normalized by GAPDH (**I**). The mRNA levels of SOCS4 were examined by RT-qPCR (**J**). Data are presented as mean ± SEM

Learning from previous studies, SOCS4 is one of the putative target genes of miR-1915-3p that functions in erythroid/megakaryocytic differentiation, and it has a complementary sequence on the 3′-UTR that can be bounded by miR-1915-3p (Fig. 2A). To further verify the binding relationship between SOCS4 and miR-1915-3p, dual luciferase reporter assay was conducted in K562 cells. The data presented that miR-1915-3p overexpression significantly decreased the luciferase activity of the reporter containing the wild-type 3′-UTR of SOCS4, when compared with the mutant form and the control (Fig. 2A-C). Overexpressing miR-1915-3p in CB-MNCs by transfection with miR-1915-3p mimics decreased the protein expression levels of SOCS4. Next, we utilized exogenous plasmid transfection to manipulate the overexpression of miR-1915-3p and examined the expression of SOCS4 in K562, Meg-01, UT-7 and TF-1 cell lines. Similar to the finding in MNCs, SOCS4 protein expression in stable-transfected pcDNA3.0-pri-miR-1915-3p-neomycin cells was considerably lower compared with that in pcDNA3.0-neomycin cells (Fig. 2D, E). Likewise, RT-qPCR results showed that miR-1915-3p overexpression decreases the mRNA levels of SOCS4 (Fig. 2F).

We have proved the expression of miR-1915-3p in the bone marrow nucleated cells in acute liver injury (ALI) mice was higher than that in normal (CON) mice on 2 days and 4 days after CCl_4_ treatment [20]. Therefore, we assessed SOCS4 protein expression in the bone marrow. Consistently the expression of SOCS4 in bone marrow cells was lower in ALI mice and even reduced to ∼ 13% compared to that of the CON mice on day 8 (Fig. 2G), suggesting that SOCS4 was one of the target genes of miR-1915-3p in vivo.

Next, we explored the effect of miR-1915-3p inhibition on the SOCS4 expression. The pcDNA3.0-miR-1915-3p-sponge-neomycin plasmid was stably transfected into K562, Meg-01, UT-7 and TF-1 cell lines, and the mRNA and protein levels of SOCS4 were analyzed (Fig. 2H-J). As expected, miR-1915-3p inhibition by its sponge improved SOCS4 expression at both mRNA and protein levels. The above result indicated that SOCS4 is one of the direct targets of miR-1915-3p.

### SOCS4 gene silencing retards erythroid differentiation process but promotes megakaryocytic differentiation

**Fig. 3:**
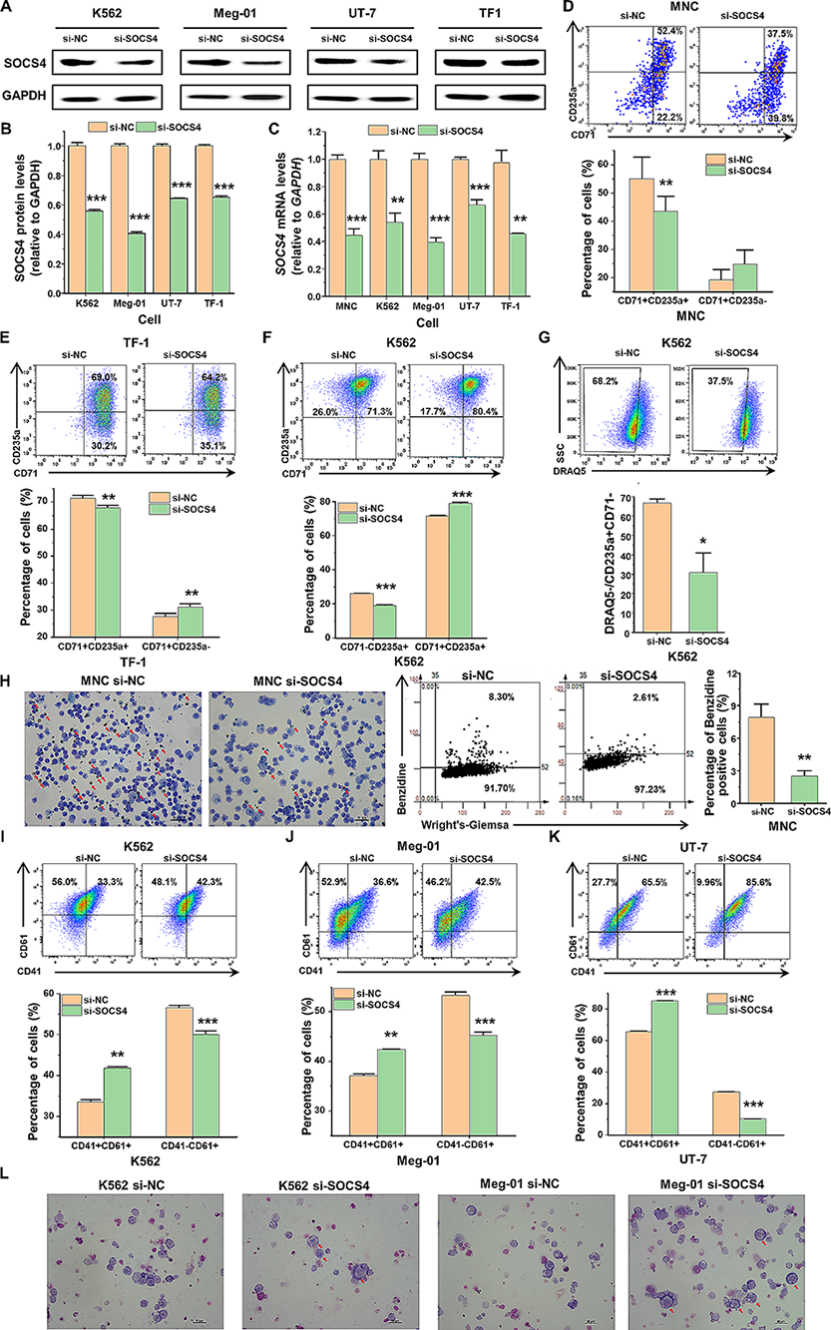
Downregulation of SOCS4 inhibits erythroid differentiation while promoting megakaryocytic differentiation. (**A**-**C**) Detection of SOCS4 expression under SOCS4 gene downregulation. SOCS4 specific siRNA or negative control (NC) siRNA were transfected into the MNCs, K562, Meg-01, UT-7 and TF-1 cells. The protein levels of SOCS4 were analyzed by Western blot (**A**), and normalized by GAPDH (**B**). The mRNA levels of SOCS4 were examined by RT-qPCR (**C**). (**D**) Detection of MNC erythroid differentiation under SOCS4 downregulation. MNCs were transfected with SOCS4 siRNA or NC siRNA, then cultured in an erythroid differentiation medium. Flow cytometry was used to examine the expression of surface marker CD71 and CD235a on day 5. (**E**) Detection of TF-1 cell erythroid differentiation under SOCS4 downregulation. TF-1 cells were transfected with SOCS4 siRNA or NC siRNA and then induced toward erythroid cells. CD71 and CD235a were examined by flow cytometry. (**F**, **G**) Detection of K562 cell erythroid differentiation under SOCS4 downregulation. K562 cells were transfected with SOCS4 siRNA or NC siRNA, then induced to erythroid differentiation and enucleation. Flow cytometry was used to examine the expression of CD71 and CD235a (**F**) and the proportion of enucleated cells (DRAQ5 negative cells, **G**). (**H**) CB-MNCs were induced for erythroid differentiation, and performed to Hemoglobin content and cell morphology detection by Benzidine staining and Wright’s‑Giemsa staining on Day 14. Middle panels are plots of quantified benzidine positive cells. Right panel is the statistical analysis of percentage of benzidine positive cells after SOCS4 silence with siRNA. (**I**-**K**) Detection of leukemia cell K562, Meg-01 and UT-7 megakaryocytic differentiation under SOCS4 downregulation. K562 (**I**), Meg-01 (**J**) and UT-7 (**K**) cells were transfected with SOCS4 siRNA or NC siRNA, then induced to megakaryocyte. CD41 and CD61 were examined by flow cytometry on day 3. (**L**) Wright’s‑Giemsa staining of K562 and Meg-01 cells in the context of SOCS4 knockdown by siRNA after PMA treatment for 3 d. Data are presented as mean ± SEM. The scale bar is 50 μm

SOCS4 protein belongs to the suppressor of cytokine signaling (SOCS) family, also known as STAT-induced STAT inhibitor protein family, which involves in the regulation of cytokine signaling [27]. There is no described biological role for SOCS4, despite broad expression in the hematopoietic system. Taken together with the results that SOCS4 mRNA level decreased during megakaryocytic differentiation and increased during erythroid differentiation, we speculated that this molecule is involved in the MEP differentiation regulation. To confirm the hypothesis, SOCS4 specific siRNA mimics was transfected into MNCs, K562, Meg01, UT-7 and TF-1 cells, and the silencing effect of SOCS4 was examined. To directly validate that the effect of transient transfection could be maintained during the induction period, K562 cells were transfected with fluorescent siRNA (FAM-siRNA), and the persistence of fluorescence was analyzed by flow cytometry every other day. Although the fluorescence intensity decreased with time, the effect remained until day 6 (Supplementary Fig. 2A). Also, SOCS4 mRNA levels of the cells transfected with SOCS4 siRNA were examined by RT-qPCR. Till day 8 post transfection, SOCS4 expression could still be down-regulated by its specific siRNA mimics (Supplementary Fig. 2B). After siRNA transfection, both the mRNA and protein levels of SOCS4 in K562, Meg01, UT-7 and TF-1 cells were markedly reduced (Fig. 3A-C). Furthermore, we also observed that MNCs in response to SOCS4 knockdown developed a decreased percentage of CD71^+^CD235a^+^ cells during erythroid induction (Fig. 3D), it indicated that erythrogenesis was blocked. A similar situation was observed in K562 and TF-1 cells during erythroid differentiation in vitro. SOCS4 silencing significantly reduced CD71^+^CD235a^+^ cells percentage in TF-1 (Fig. 3E) and CD71^−^CD235a^+^ cells percentage and enucleation ratio (DRAQ5^−^_/_CD235a^+^ cells) in K562 cells (Fig. 3F, G). Benzidine staining was performed to identify hemoglobin-containing cells, the present of which was an indicator of erythrocyte differentiation. The results demonstrated that SOCS4 downregulation decreased the hemoglobin-containing cells and reduced the cell volume, as well as decreased nuclear shrinkage and incidence of a lopsided nucleus under erythrocyte differentiation (Fig. 3H, Supplementary Fig. 3).

To confirm the role of SOCS4 in megakaryopoiesis, human leukemia cell lines, K562, Meg-01 and UT-7 cells were transfected with siRNA mimics against SOCS4 and treated with PMA to induce into megakaryocyte. A remarkable increase of CD41^+^CD61^+^ percentage was observed in cells transfected with SOCS4 siRNA compared with cells transfected with NC siRNA (Fig. 3I-K). Wright-Giemsa staining demonstrated more polyploid cells and larger cell dimensions (red arrows) with SOCS4 knockdown (Fig. 3L). These data suggested that SOCS4 silencing promoted megakaryocytic differentiation and inhibited erythroid differentiation.

### Overexpression of SOCS4 inhibits megakaryopoiesis and reverses the miR-1915-3p-induced erythroid differentiation blockage

**Fig. 4:**
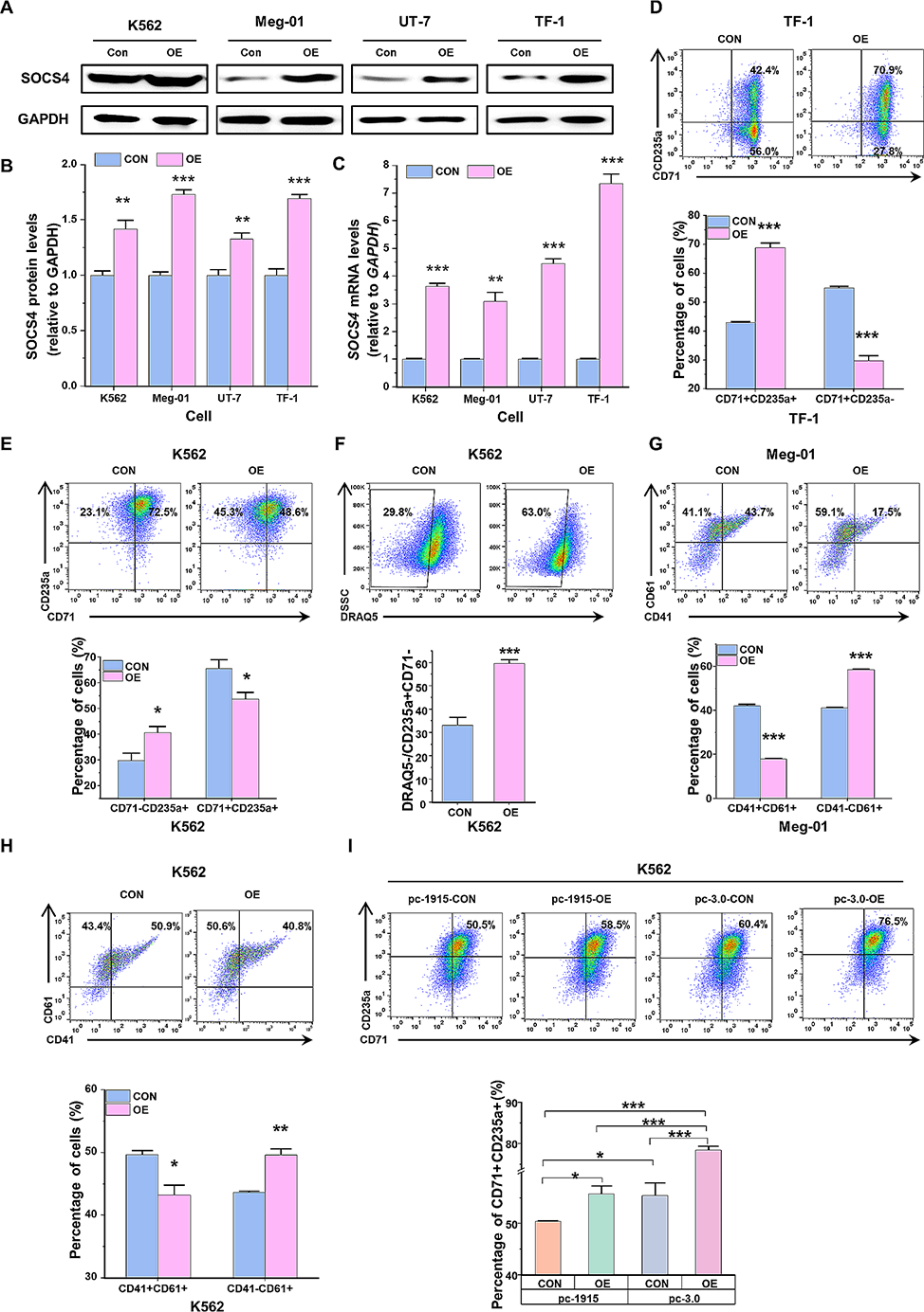
SOCS4 overexpression (OE) induces erythroid differentiation, but reduces megakaryocytic differentiation. (**A**-**C**) Detection of SOCS4 expression under SOCS4 gene upregulation. pcDNA3.0-SOCS4-neomycin plasmid or pcDNA3.0-neomycin control plasmid were transfected into the K562, Meg-01, UT-7 and TF-1 cells. Stable overexpressed cell lines were selected using G418. The protein levels of SOCS4 were analyzed by Western blot (**A**), and normalized by GAPDH (**B**). The mRNA levels of SOCS4 were examined by RT-qPCR (**C**). (**D**) Detection of TF-1 cell erythroid differentiation under SOCS4 upregulation. The stable SOCS4 overexpressed TF-1 cells were induced to erythroid differentiation and the expression of CD71 and CD235a were examined by flow cytometry. (**E**, **F**) Detection of K562 cell erythroid differentiation under SOCS4 upregulation. Stable SOCS4 overexpressed K562 cells were induced to erythroid differentiation and enucleation, flow cytometry was used to examine CD71 and CD235a (**E**) and the proportion of enucleated cells (DRAQ5 negative cells, **F**). (**G**, **H**) Detection of leukemia cell Meg-01 and K562 megakaryocytic differentiation under SOCS4 upregulation. Stable SOCS4 overexpressed Meg-01 cells (**G**) and K562 cells (**H**) were induced to megakaryocyte. CD41 and CD61 were examined by flow cytometry. (**I**) Detection of K562 cell erythroid differentiation under SOCS4 overexpression and miR-1915-3p upregulation. Stable miR-1915-3p overexpressing K562 cells were transiently transfected with pcDNA3.0-SOCS4-neomycin (or pcDNA3.0-neomycin control plasmid), then induced to erythroid differentiation. CD71 and CD235a were examined by flow cytometry on day 4. Data are presented as mean ± SEM

To further elucidate whether SOCS4 overexpression induce erythroid differentiation and suppress megakaryopoiesis, the expression plasmid pcDNA3.0-SOCS4-neomycin was constructed and transfected into K562, Meg-01, UT-7 and TF-1 cells. The upregulation of SOCS4 was confirmed at both the mRNA and protein levels (Fig. 4A-C). SOCS4 overexpression significantly promoted erythroid differentiation of TF-1 and K562 cells, as assessed by the CD71^+^CD235a^+^ percentage and enucleated rate (Fig. 4D-F). We next examined the effect of upregulating SOCS4 during megakaryocytic differentiation. Both in K562 and Meg-01 cells, the percentage of CD41^+^CD61^+^ cells were dropped markedly in response to SOCS4 overexpression (Fig. 4G, H).

In addition, we examined whether miR-1915-3p-induced erythroid differentiation blockage could be antagonized by upregulating SOCS4 expression. In the K562 cells, an exogenous increase of SOCS4 rescued the decline of erythroid surface markers which was resulted from miR-1915-3p transfection (Fig. 4I). It indicated that the anti-erythroid differentiation effects of miR-1915-3p were partially abrogated by the concomitant SOCS4 overexpression. In summary, miR-1915-3p may promote megakaryopoiesis and inhibit erythroid differentiation by regulating SOCS4 expression.

## Discussion

In this report, we revealed that miR-1915-3p was able to alter MEP differentiation fate and inhibit erythroid cell production to promote megakaryoblast differentiation. The target genes of miR-1915-3p were scanned by mRNA expression profiling and bioinformatic analysis. Then the dual-luciferase reporter assay confirmed that miR-1915-3p could directly interact with SOCS4 3′-UTR and suppress the expression of SOCS4. We have subsequently utilized gain- and lost-of-function studies to further define the role of miR-1915-3p/SOCS4 axis in the fate selection of MEPs.

There are two major differentiation pathways of MEPs, megakaryocytic and erythroid differentiation, in which miRNA plays an important regulatory role [28]. In previous studies, some miRNAs, such as miR-146b-5p [29] and miR-125b [13, 14, 17], promoting both erythroid and megakaryocytic differentiation, do not demonstrate lineage bias. But others, like miR-1915-3p in this study and miR-150 [16] showed a preference to megakaryocytic differentiation by inhibiting erythrocytic differentiation. Further study on the role of the same miRNA in different hematopoietic lineages will help us to control differentiation more specifically.

An increasing number of studies have shown that miR-1915-3p plays significant roles in tumor development and inflammatory immunity processes. miR-1915-3p functioned as a tumor suppressor and blocked tumor progression by regulating several signaling pathways in digestive system tumors [24, 30]. miR-1915-3p modulated the development of gastric cancer and colorectal cancer through the repression of RAGE, NFIX, and BCL-2 [24, 31, 32]. Although miRNAs during human erythroid and megakaryocytic differentiation have previously been profiled by global transcriptome analysis [26, 33–35], the role of miR-1915-3p remains largely unrevealed and unproven in hematopoietic differentiation, which may be due to the late discovery and high GC content of miR-1915-3p. We rechecked the data of miRNA profile during human erythropoiesis in our previous study [34] and found that the expression of miR-1915-3p was down-regulated during erythroid differentiation, consistent with the discovery in this study. However, because of its undetectable expression in some erythroid stages, miR-1915-3p was not considered as the miRNAs with significant differential expression during erythropoiesis in that study.

Our previous findings firstly revealed the distinct role of miR-1915-3p in regulating thrombopoiesis. After the injection of PMPs with enhanced miR-1915-3p into irradiated mice, higher proportions of whole megakaryocytic cells and MEPs in the bone marrow and an increased count of platelets in the peripheral blood were detected. However, in the previous study, the number of red blood cells was not found to be altered [20]. A possible reason might be the complicated content of PMPs. In this study, we further revealed the role of miR-1915-3p in erythroid differentiation and the mechanisms underlying. Enhancing the expression of miR-1915-3p in HSPCs inhibits the cells to erythroid differentiation, to drive the cells toward megakaryocytic fate and produce platelets. The complete reverse of lineage bias was not detected under miRNA modification, multiple targets of miR-1915-3p resulted mild effect of the miRNA, or partial gene modification due to the current gene modification methods, might be the reason of partial biological effects of miR-1915-3p detected in this study.

SOCS4 belongs to the SOCS family, known as cytokine-inducible negative regulators of cytokine signaling, including 8 members of SOCS1-SOCS7 and CIS (proteins having SH2 structural domain induced by cytokine). It has been reported that SOCS family proteins play important roles in cell proliferation, development, differentiation, and homeostasis [36, 37]. Three of the SOCS family proteins thus far identified have been shown to play important roles in megakaryocytic and erythroid differentiation (CIS, SOCS1, and SOCS3). SOCS1 inhibits TPO signaling of megakaryopoiesis [38]. SOCS3 binds to the EPO receptor and JAK2 and acts as a negative regulator of fetal liver erythropoiesis. In mouse model, SOCS3 deletion results in an embryonic lethality at 12-16 days, associated with marked erythrocytosis [39, 40]. Similar to SOCS3, CIS can bind to EPO receptor, and its overexpression in transiently transfected cells blocks EPO signaling. However, CIS-deficient mice are phenotypically normal in all regards [41]. In this study, we found that SOCS4 inhibits erythroid differentiation and promotes megakaryocytic differentiation in leukemia cell lines independently of TPO or EPO, but its role in the presence of cytokines in primary cells needs to be further explored, and the related molecular mechanisms still need to be further revealed.

## Conclusions

Taken together, our findings contribute to elucidating the specific effect of miR-1915-3p and SOCS4 in megakaryocytic and erythroid differentiation. miR-1915-3p acts as a negative regulator of erythropoiesis, and a supporter for the megakaryocytic fate. The effect on differentiation is, at least partially, mediated by the downregulation of SOCS4. The application of miR-1915-3p or small molecules that regulate its downstream signaling pathway might provide potential strategies for anemia and thrombocytosis in individuals.

### Electronic supplementary material

Below is the link to the electronic supplementary material.

Supplementary Material 1

## Data Availability

No datasets were generated or analysed during the current study.

## Abbreviations

CB-MNC: Human cord blood mononuclear cell
EPO: Erythropoietin
HSPC: Hematopoietic stem/progenitor cell
MEP: Megakaryotic and erythroid progenitor cell
MP: Microparticle
PMP: Platelet-derived microparticle
TPO: Thrombopoietin
SOCS: Suppressor of cytokine signaling
