# Supplementary material for: miR-1915-3p regulates megakaryocytic and erythroid differentiation by targeting SOCS4
Source: Thromb J. 2024 Aug 9;22:74. doi: 10.1186/s12959-024-00615-6 (PMC11316338; doi:10.1186/s12959-024-00615-6)

**Supplementary Information**

**Supplementary Table 1.**

Primers used in RT-qPCR.

| Primer name | Primer Sequence (5’-3’) | Annealing temperature (^o^C) |
| --- | --- | --- |
| miR-1915-3p | CCCCAGGGCGACGCGGCGGG | 60 ^o^C |
| U6 | CGCTTCGGCAGCACATATACTA | 60 ^o^C |
| U3 | CGCTTCGGCAGCACATATACTA | 60 ^o^C |
| FAM19A2 | Forward: TGTTAAAACGGGAACTTGTGAGG | 58 ^o^C |
|  | Reverse: AAGCATCCACACATGATGGAG |  |
| CLCN6 | Forward: TCCTTGGAGTGCTGTTCAGTG | 58 ^o^C |
|  | Reverse: CTTGTCTCTGTCGCTTCGGA |  |
| SOCS4 | Forward: GCCGACAGAAAAGACGGTTAT | 58 ^o^C |
|  | Reverse: GGTTCCTTAAAGACACTTCGGTT |  |
| GAPDH | Forward: GAGTCAACGGATTTGGTCGT | 58 ^o^C |
|  | Reverse: TTGATTTTGGAGGGATCTCG |  |

U3 is the universal downstream primer of miRNA.

**Supplementary Figure 1.**


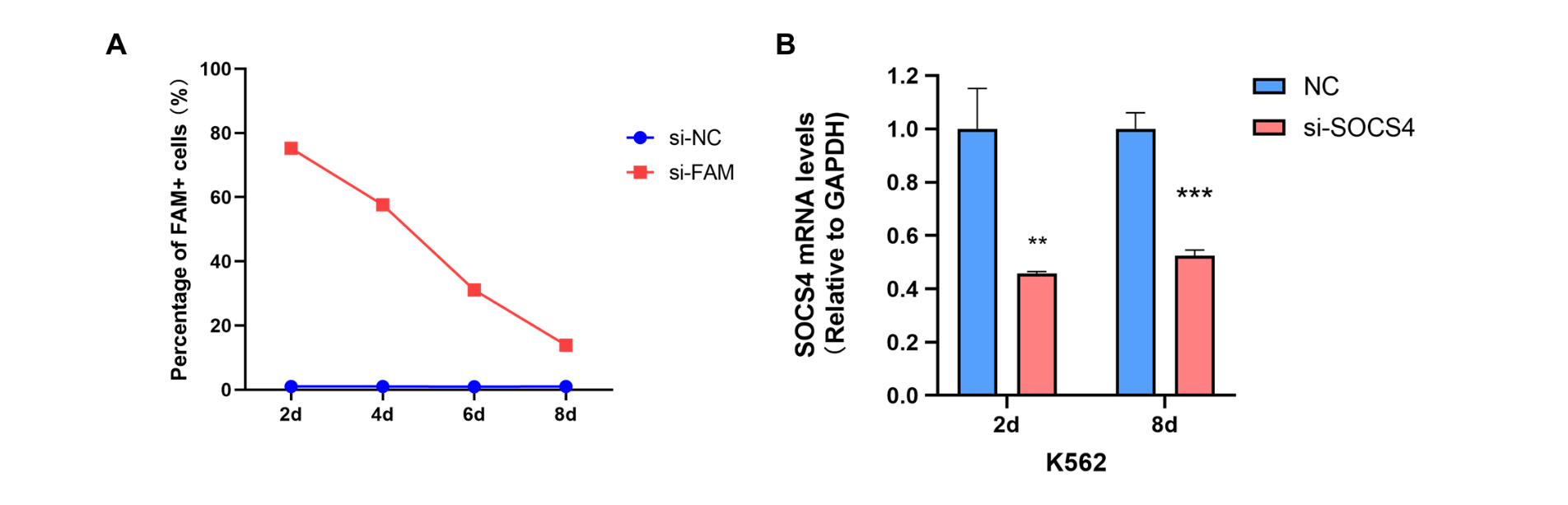


**Fig. S1 Duration of silencing effect after transfection with siRNA.**

(A) The fluorescent siRNA (FAM-siRNA) or negative control siRNA (NC-siRNA) were transfected into K562 cells, and the fluorescence was analyzed by flow cytometry every other day. (B) SOCS4 specific siRNA or negative control siRNA (NC-siRNA) were transfected into K562 cells. The mRNA levels of SOCS4 were examined by RT-qPCR on Days 2 and 8 after transfection. Data are presented as mean ± SEM.

**Supplementary Figure 2.**


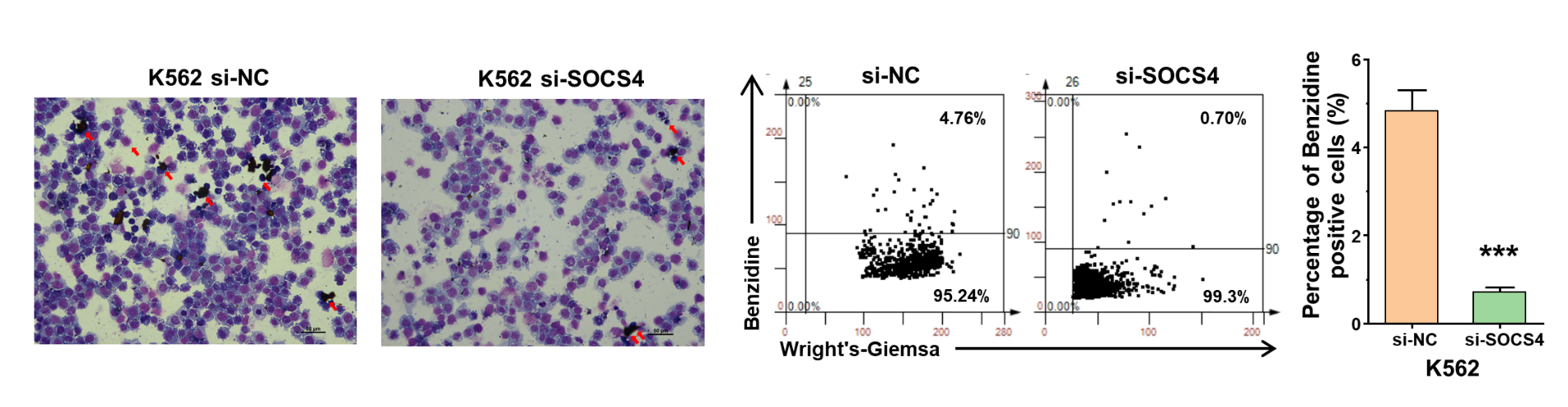


**Fig. S2 Hemoglobin expression and cell morphology of K562 during erythropoiesis.**

K562 cells were induced to erythroid differentiation for 8 days, and performed to Hemoglobin expression and cell morphology detection by Benzidine staining and Wright's‑Giemsa staining. Middle panels are plots of quantified benzidine positive cells. Right panel is the statistical analysis of percentage of benzidine positive cells after SOCS4 silence with siRNA.

**Supplementary Figure 3.** The original source images of Western blot related to Figure 2D.


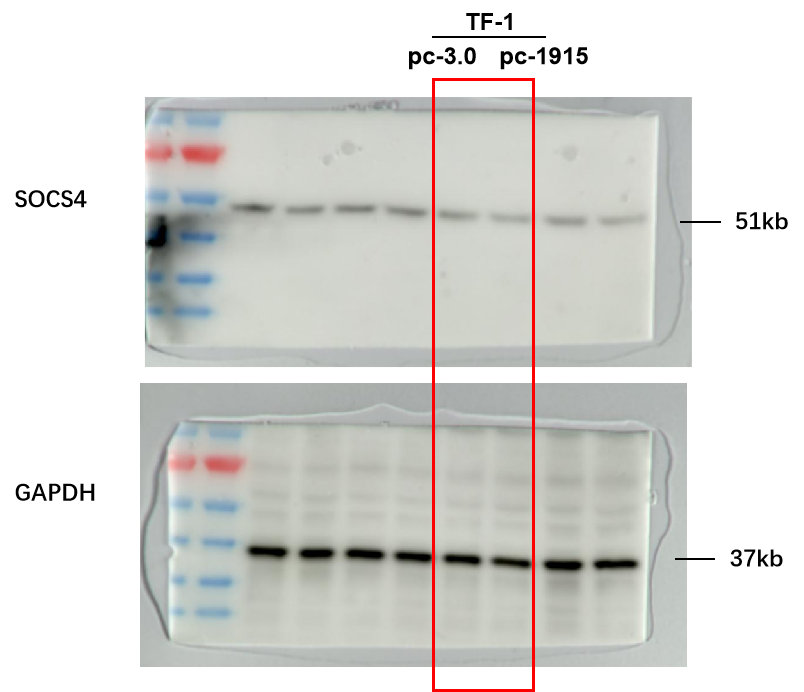

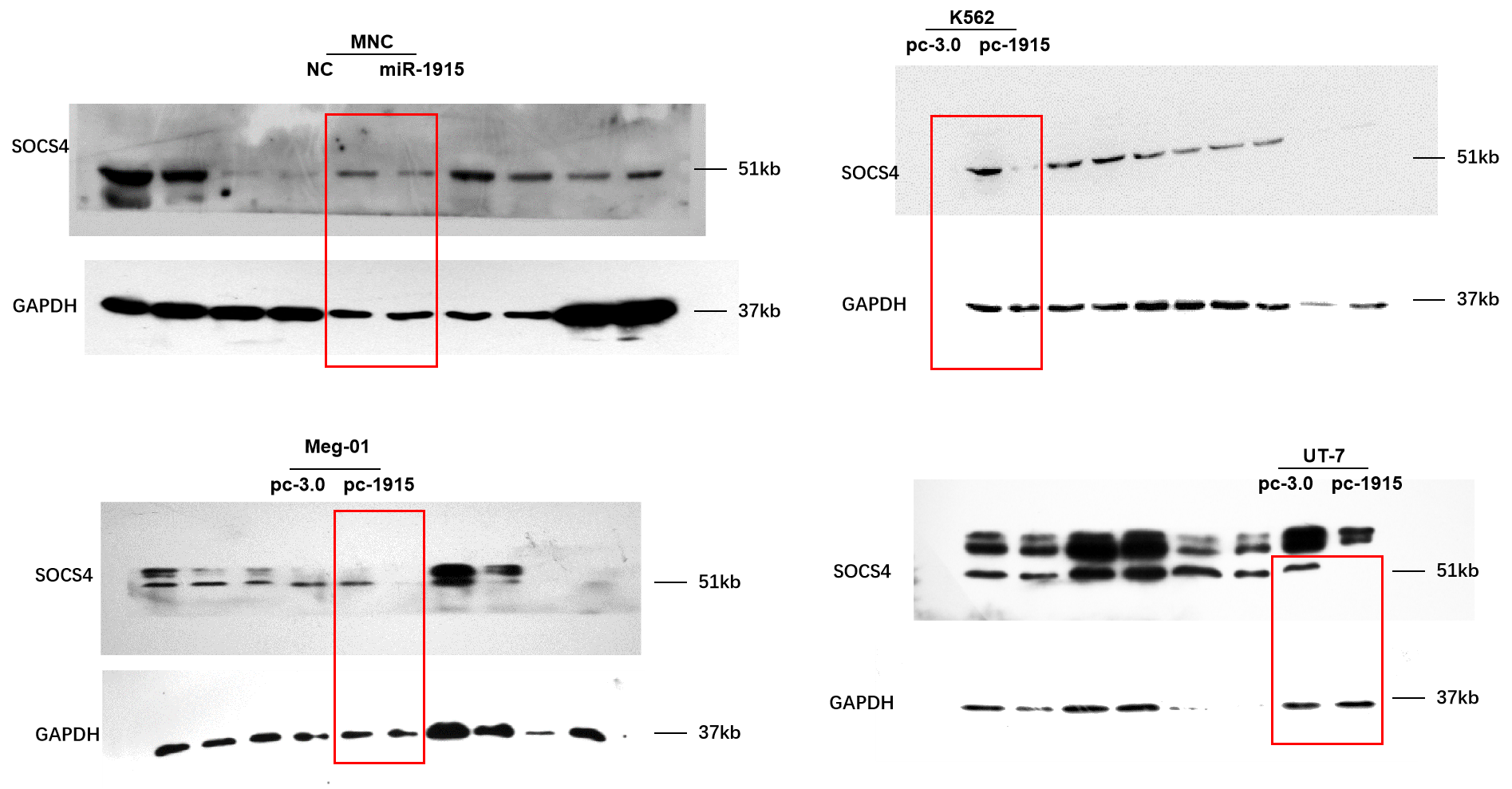


**Supplementary Figure 4.** The original source images of Western blot related to Figure 2H.


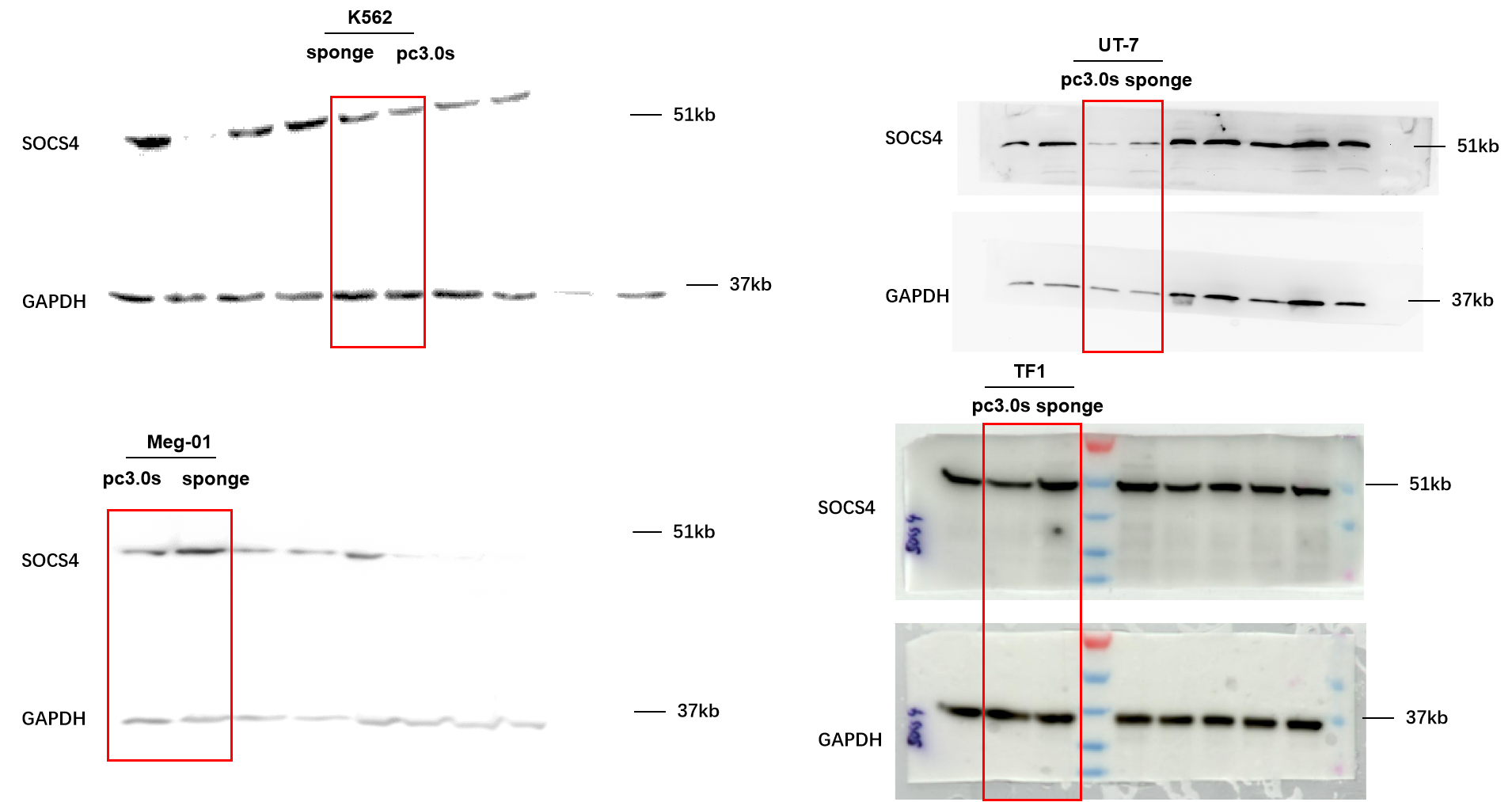


**Supplementary Figure 5.** The original source images of Western blot related to Figure 3A.


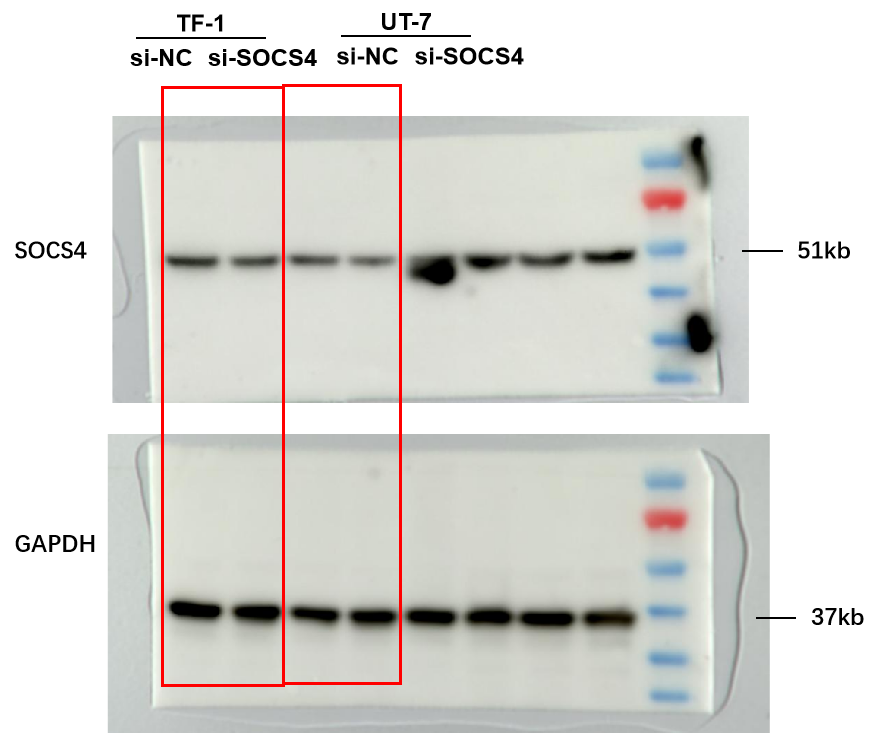

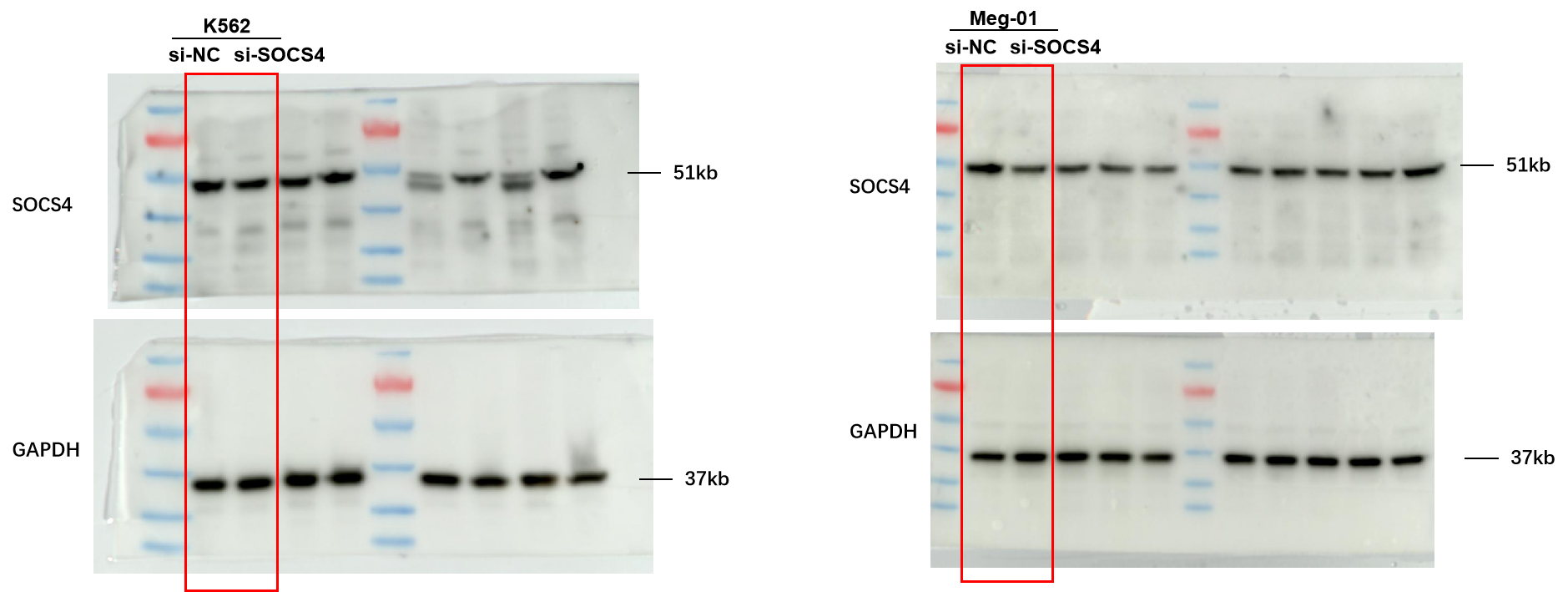


**Supplementary Figure 6.** The original source images of Western blot related to Figure 4A.


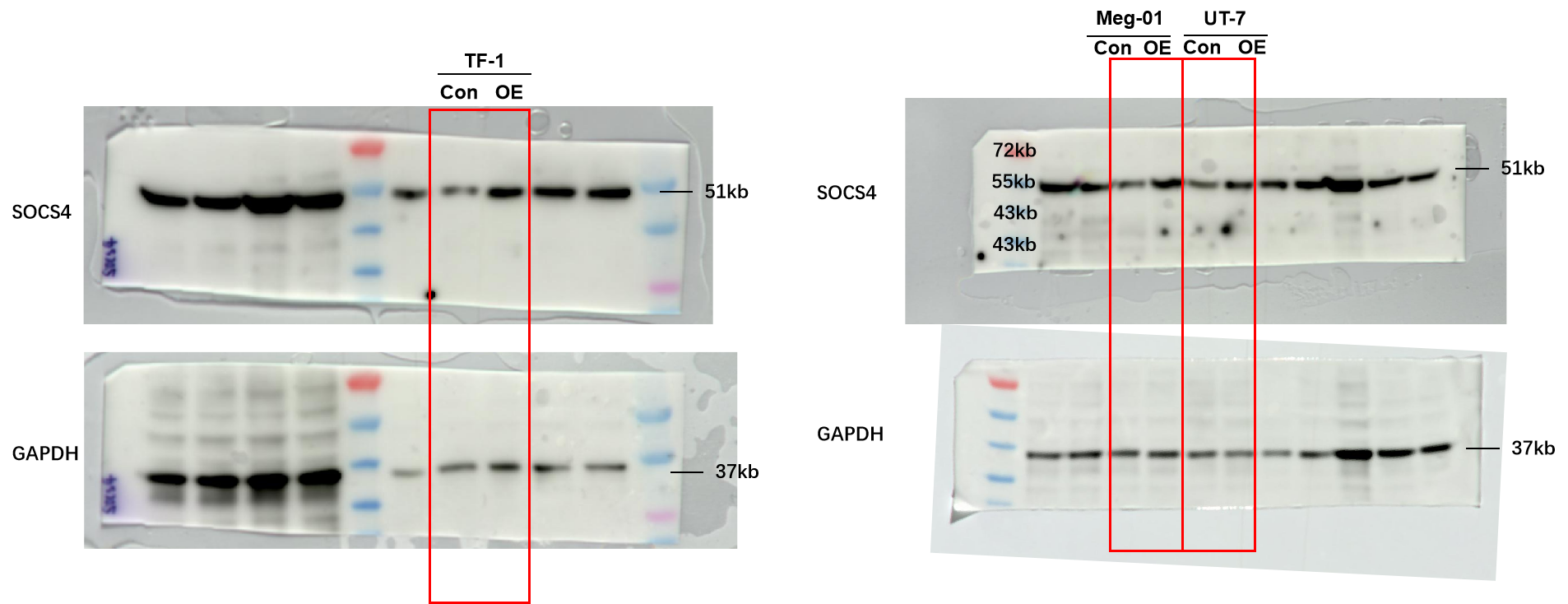

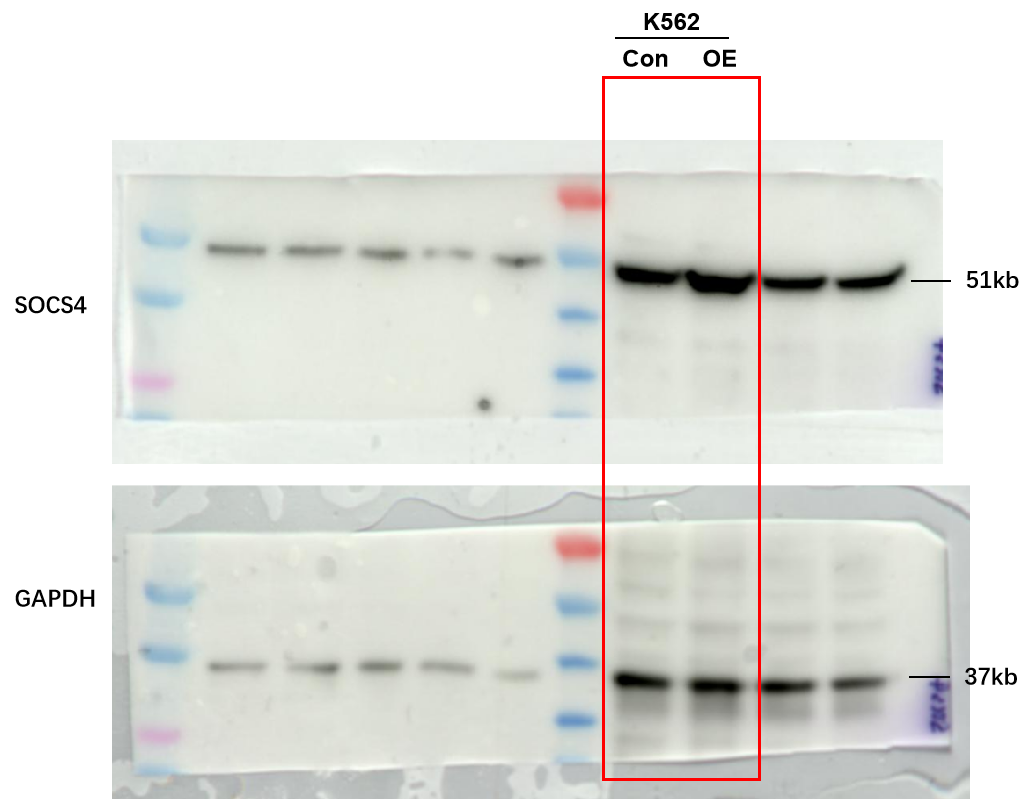

Supplement: Supplementary file 1 — Supplementary Material 1 [file 12959_2024_615_MOESM1_ESM.docx]
